# A Case of Antimitochondrial Antibody Negative Primary Biliary Cirrhosis from Bangladesh and Review of Literature

**DOI:** 10.5005/jp-journals-10018-1150

**Published:** 2016-07-09

**Authors:** Roksana Begum, Mamun-Al Mahtab, Ayub Al Mamun, Biplob Kumar Saha, Sharker Mohammad Shahadat Hossain, Dulal Chandra Das, Sheikh Mohammad Fazle Akbar, Mohammad Kamal, Salimur Rahman

**Affiliations:** 1 Department of Hepatology, Bangabandhu Sheikh Mujib Medical University, Dhaka, Bangladesh; 2 Department of Medical Sciences, Toshiba General Hospital, Tokyo, Japan; 3 Department of Pathology, Bangabandhu Sheikh Mujib Medical University, Dhaka, Bangladesh

**Keywords:** Antimitochondrial antibody negative, Primary biliary cirrhosis.

## Abstract

Primary biliary cirrhosis (PBC) is very closely associated with the presence of antimitochondrial antibodies (AMA). Nevertheless, 5 to 10% of patients with typical features of primary biliary cirrhosis do not have detectable AMA. They are referred to as AMA negative PBC. We report a case of PBC who was AMA-negative. The patient was a middle-aged woman seeking help of dermatologist for her complaint of itching. The diagnosis was confirmed by histopathology, whereas other possible causes of cirrhosis were excluded.

**How to cite this article:**

Begum R, Mahtab MA, Mamun AA, Saha BK, Hossain SMS, Saha DC, Akbar SMF, Kamal M, Rahman S. A Case of Antimitochondrial Antibody Negative Primary Biliary Cirrhosis from Bangladesh and Review of Literature. Euroasian J Hepato-Gastroenterol 2015;5(2):122-126.

## INTRODUCTION

Primary biliary cirrhosis (PBC) is a chronic, progressive autoimmune liver disease that is characterized by nonsuppurative destruction of intrahepatic ducts, resulting in cholestasis, cirrhosis, and end-stage liver disease.^1,2^ The incidence and prevalence of PBC have been reported from multiple European countries, North America, Australia, Israel, India and Japan.^3^ The annual incidence rates ranged from 0.7 to 49 cases per million. Although there have been an increasing number of reported diagnoses, PBC is still thought to be very rare in Asian populations. Moreover, there is a lack of solid epidemiological information relevant to this issue. Till date there is only one case report of PBC from Bangladesh who was antimitochon-drial antibodies (AMA) positive. We report a 31-year-old woman of PBC who is AMA negative and provide a brief review of the literature on AMA-negative PBC.

## CASE REPORT

A 31 years old woman, diabetic for 2 years was admitted to Bangabandhu Sheikh Mujib Medical University, Dhaka, Bangladesh for generalized itching, progressively increasing fatigue and weight loss for 18 months. The patient did not give previous history of Jaundice, pale stool, upper abdominal pain, fever, altered bowel habit, heat or cold intolerance and any nodular swelling at any parts of body or abdomen.

On physical examination, the patient was mildly anemic, nonicteric, scratch mark all over the body with mild hepatomegaly. No stigmata of chronic liver disease were found. We investigated her extensively.

A routine blood test revealed Hb -9.0 gm/dL, ESR -95 mm in first hour, and total count of WBC -9 × 109/L (neutrophils -70%, lymphocytes -22%, eosinophils 2% and monocytes 6%). Her liver profile showed serum bilirubin 1.4 mg/dl (normal, 0.3-1.2 mg/dl); alkaline phosphatase 557 U/L (normal, 50-136 U/L); Gamma GT 184U/L (normal up to -39U/L), AST -46 U/L, ALT 25 U/L normal, prothrombin time was 12.8/12 seconds with INR -1.07 and serum albumin 3.5 gm/dl. Her total serum cholesterol was -217 mg/dl, HDL -37 mg/dl, LDL 146 g/dl and TG 170 mg/dl. Antinuclear antibody (ANA) was positive, serum immunoglobulin G (IgG) and IgM were minimally elevated. Serum thyroid stimulating hormone (TSH) was 1.385 mlU/ml. Serology for AMA, Hepatitis B surface antigen (HBsAg), anti Hepatitis C virus (HCV) Antibody, anti-ds DNA, anti LKM-1 and anti-smooth muscle Ab was negative. Anti HBc total was positive but Hepattis B virus (HBV) DNA was undetected by polymerase chain reaction (PCR) method. Endoscopy of upper gastrointestinal tract (GIT) showed gastric erosion.

**Fig. 1:**
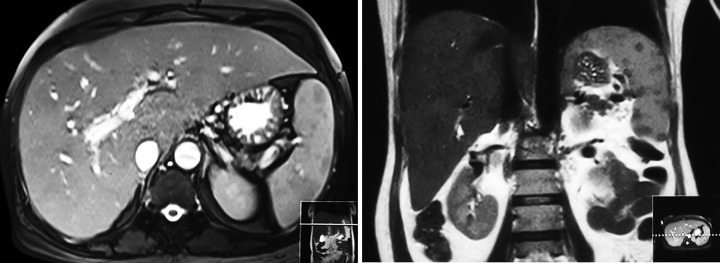
Magnetic resonance cholangiopancreatography showing normal biliary tree and main pancreatic duct (MPD)

Abdominal ultrasonography showed liver is mildly enlarged in size with coarse echotexture. Spleen was also mildly enlarged. We also performed magnetic resonance cholangio-pancreatography (MRCP) to exclude any co-existent obstructive etiology, and found normal biliary tree (Fig. 1).

**Fig. 2:**
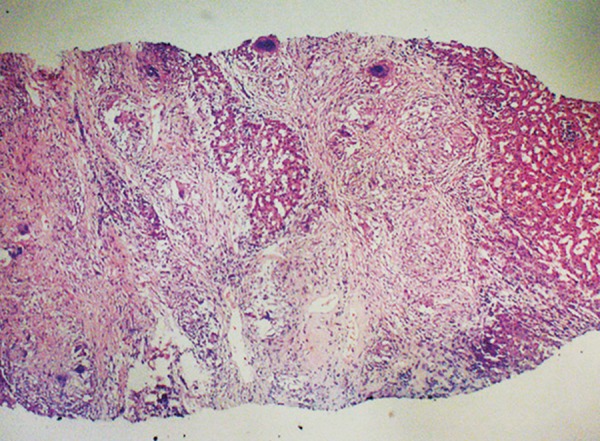
The portal zone contains well filled granuloma. Adjacent bile duct shows damage

**Fig. 3:**
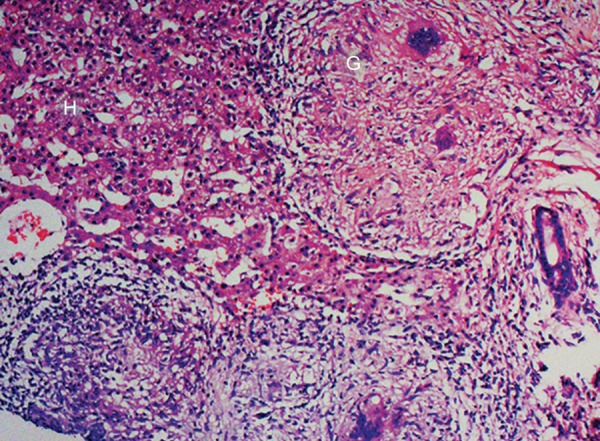
There is scarring and septa contains lymphoid aggregates. Bile ducts are rare and inconspicuous

**Fig. 4:**
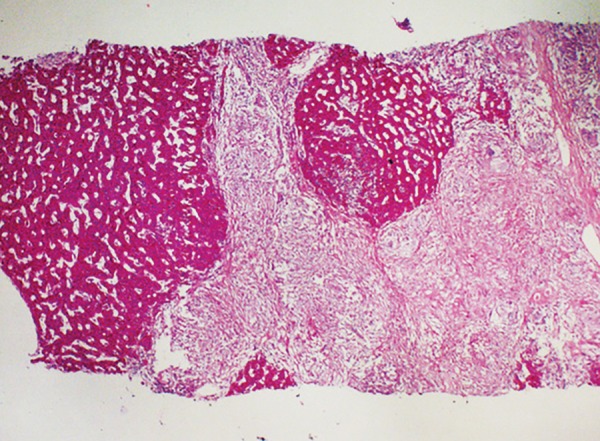
Aggregates of lymphoid cells

A subsequent liver biopsy was performed percuta-neously under local anesthesia with full aseptic precautions. A core of the liver tissue was obtained and sent for histopathology to a histopathologist who was unaware of the clinical and laboratory findings of the patient. Histological features showed thick irregular fibrous bands separating the portal areas. These contain many noncaseating granulomas, a moderate number of chronic inflammatory cells and small number of acute inflammatory cells. Bile ducts have been largely disappeared. Those present show inflammatory cells infiltrate in the walls. The hepatocytes are unremarkable. Features are consistent with primary biliary cirrhosis stage-3 (Figs 2 to 4).

We, thus, concluded that the patient is suffering from AMA negative PBC. The patient is now receiving symptomatic treatment with cholestyramine, ursodeoxycholic acid (UDCA), probiotics, lactulose and antiulcer drugs.

### Discussion and Review of Literature

Primary biliary cirrhosis has been considered a disease of middle-aged Caucasian women. The patient is usually 40 to 60 years of age, but can be as old as 80 years or as young as 15 years. Ten percent are male, in whom the disease runs a similar course.^4^ Because most patients with PBC are of Caucasian ethnicity, data on the clinical presentation and disease severity in other ethnic groups are limited.

A large, prospective, randomized, US-based multi-center trial conducted by Peters et al^5^ that included 535 patients with PBC showed a similar mean age (52.1 ± 9.5 *vs* 51.5 ± 9.4 years), female to male predominance (13.9:1 *vs* 12.2:1), and AMA-positive rates (92.4 *vs* 86.3%) between Caucasian and noncaucasian (African American and Hispanic) populations, respectively.

The manifestation of PBC among Asian patients is less understood given the infrequency of the disease in this population. In a recent report from Singapore, overall survival free of liver transplantation at 5 years was near 90%, which is significantly better than that quoted in European and North American studies. However, a direct comparison among studies is not possible, and prior studies from Asian countries demonstrated 5-year survival rates near 70 to 80%, more consistent with the European experience.^3^

The etiological factors triggering the production of autoantibodies in PBC remain enigmatic. Environmental and genetic factors have been shown to influence susceptibility to PBC. Currently, there is evidence to support the role of infectious agents, such as bacteria, retroviruses, and xenobiotics, in the pathogenesis of PBC.^6^ Xenobiotics are foreign compounds that may complex to self proteins. Infectious agents are *E. coli, Chlamydia pneumoniae, Lactobacillus,* and *Helicobacter pylori^3^* It is thought that exposure to these agents, particularly xenobiotics, can alter the structure of native proteins, inducing an immune response to self-proteins, a phenomenon referred to as molecular mimicry.^7^

Identification of several PBC clusters lends anecdotal support to this theory, as multiple cases of PBC have been reported within a single family, both among genetically related individuals and genetically unrelated individuals living in the same household.^8^ In addition, a high prevalence of PBC has been observed in certain geographic areas.^9,10^ One study from the United Kingdom and Greece reported that PBC clusters have often been identified in areas close to water reservoirs, coal mining areas, and toxic waste disposal sites, adding strength to the notion that environmental factors may serve as a catalyst to PBC.^10^ Several studies have identified a significant risk among persons genetically related to patients with PBC compared with the risk noted in the general popu-lation.^11,12^ Studies examining the disease association with major histocompatibility complex class II molecules have identified HLA-DR8, DRB1*08, DR3, DPB1*0301, DR2, DPB1*0501, and DRB1*0803 as risk factors for PBC.^4^ Significant genetic associations with PBC include polymorphisms in the tumor necrosis factor-a promoter region, the TAP1/TAP2 genes on chromosome 6, the promoter region of the IL-10 gene, the natural resistance-associated macrophage protein 1 gene, and the vitamin D receptor gene.^4^

Fatigue and pruritus are by far the most common symptoms reported by the patient with PBC. Jaundice, on the other hand, is a late event and is associated with a poor prognosis. Right upper quadrant abdominal pain is reported in 10% of patients.^3^ On examination, patients may be mildly anemic, with firm hepatomegaly and just palpable spleen. Primary biliary cirrhosis may be associated with a number of diseases, in particular, autoimmune diseases such as rheumatoid arthritis, dermatomyositis, mixed connective tissue disease and systemic lupus erythematosus, autoimmune thyroiditis, and ulcerative colitis^13^ has been reported PBC. There is a small increase in overall cancer risk and mortality in PBC patients.^14^

According to the American Association for the Study of Liver Diseases (AASLD) guidelines, PBC should be considered in patients with elevated serum ALP levels and the diagnosis is established if two of the following three criteria are met: AMA is detected; elevated ALP levels are indicative of cholestasis; and a liver histology confirms the nonsuppurative destruction of intrahepatic ducts.^16^ With the sensitivity and specificity of AMAs approaching 95%, detection of AMA is important in the diagnosis of PBC.

However, the diagnosis of AMA-negative PBC requires a liver biopsy that demonstrates the typical features of bile duct destruction seen in PBC. The diagnosis is more certain if granulomas are present. Immu-noglobulin M levels are lower in AMA-negative than AMA-positive patients with PBC. A recent meta-analysis has examined published reports of patients treated for AMA-negative PBC, which only totaled 52 patients. The authors concluded no difference in biochemical response to UDCA was observed when patients with AMA-posi-tive and AMA-negative PBC were compared.^15^

A higher titer of ANA, anti-smooth muscle antibody, gamma globulin, and lower IgM have been demonstrated in patients with AMA-negative PBC compared with those with AMA-positive PBC.^15^

Once a diagnosis of PBC is established, regardless of whether it is positive or negative for AMAs, the most widely accepted treatment is UDCA. This pharmaco-therapy is the only one approved by the US Food and Drug Administration (FDA) as a treatment for PBC and the only agent endorsed by the AASLD for this purpose.^16^ Ursodeoxycholic acid is well tolerated in most patients. The most commonly reported side effects are headache, constipation and diarrhea.^17^

In Liu study,^18^ treatment of AMA-negative and AMA-positive PBC with UDCA resulted in similar improvements in ALP, IgM, and glutamyl transpeptidase levels. Both groups also showed a significant increase of regulatory T cells after a 1-year follow-up. In a retrospective analysis by Mayo Clinic researchers, the biochemical parameters and Mayo risk score for patients with AMA-negative and AMA-positive PBC were similar, and both groups demonstrated similar treatment outcomes with UDCA.^19^

Other pharmacotherapies that have been used to treat PBC include colchicine and methotrexate, but these have been prescribed off-label, as their role is controversial and their benefits are less clear.^20^ Some novel agents namely fenofibrate and benzafibrate showed incomplete response.^3^

Therefore, once UDCA ceases to control the disease and the patient progresses to end-stage liver disease, liver transplantation should be considered, regardless of the patient’s AMA status.^21^ In a study by Lee,^21^ the clinical outcomes following orthotopic liver transplantation in patients with AMA-negative PBC were examined after a median follow-up of 36 months. The authors concluded that the graft and patient survival rates and subsequent histological changes, including disease recurrence and steroid-resistant or late rejections, were comparable with those of patients with AMA-positive PBC.^14^

The causes of death in PBC include variceal hemorrhage, encephalopathy and Gram-negative septicemia.

Antimitochondrial antibody-negative and AMA-positive PBC share similar clinical, biochemical and histopathological features. Improvement in sensitivity of techniques for detecting AMAs will further bridge the gap between AMA-negative and AMA-positive PBC.^22^ Therefore, detection of AMAs alone should not influence the diagnosis or treatment of PBC, and the disease should be managed like other PBC patients.^15^

## References

[1] Kaplan MM, Gershwin ME (2005). Primary biliary cirrhosis.. N Engl J Med.

[2] Selmi C, Invernizzi P, Zuin M, Podda M, Seldin MF, Gershwin ME (2005). Genes and (auto) immunity in primary biliary cirrhosis.. Genes Immun.

[3] Boyer T, Manns M, Sanyal A, Zakim D (2012). Zakim and Boyer’s hepatology..

[4] Dooley J, Sherlock S (2011). Sherlock’s diseases of the liver and biliary system..

[5] Peters MG, Di Bisceglie AM, Kowdley KV (2007). For the Pumps Group. Differences between Caucasian, African American, and Hispanic patients with primary biliary cirrhosis in the United States.. Hepatology.

[6] Van de Water J, Ishibashi H, Coppel RL, Gershwin ME (2001). Molecular mimicry and primary biliary cirrhosis: premises not promises.. Hepatology.

[7] Long SA, van de Water J, Gershwin ME (2002). Antimitochon-drial antibodies in primary biliary cirrhosis: the role of xenobiotics.. Autoimmun Rev.

[8] Abu-Mouch S, Selmi C, Benson GD (2003). Geographic clusters of primary biliary cirrhosis.. Clin Dev Immunol.

[9] Kim WR, Poterucha JJ, Jorgensen RA (1997). Does anti-mitochondrial antibody status affect response to treatment in patients with primary biliary cirrhosis? Outcomes of ursodeoxycholic acid therapy and liver transplantation.. Hepatology.

[10] Smyk D, Mytilinaiou MG, Rigopoulou EI, Bogdanos DP (2010). Primary biliary cirrhosis triggers in water reservoirs, coal mining areas and waste disposal sites: from Newcastle to New York.. Dis Markers.

[11] Kim WR, Lindor KD, Locke GR (2000). Epidemiology and natural history of primary biliary cirrhosis in a US community.. Gastroenterology.

[12] James OFW, Bhopal R, Howl D, Gray J, Burt AD, Metcalf JV (1999). Primary biliary cirrhosis once rare, now common in the United Kingdom?. Hepatology.

[13] Hall S (1984). Systemic Lupus Erythematosus developing in patients with primary biliary cirrhosis.. Ann Int Med.

[14] Howel D, Metcalf J, Gray J, Newman W, Jones D, James O (1999). Cancer risk in primary biliary cirrhosis: a study in northern England.. Gut.

[15] Lindor KD, Gershwin ME, Poupon R (2009). American association for study of liver diseases. Primary biliary cirrhosis.. Hepatology.

[16] Bassendine MF, Yeaman SJ (1992). Serological markers of primary biliary cirrhosis: diagnosis, prognosis and subsets.. Hepatology.

[17] Siegel JL, Jorgensen R, Angulo P, Lindor KD (2003). Treatment with ursodeoxycholic acid is associated with weight gain in patients with primary biliary cirrhosis.. J Clin Gastro- enterol.

[18] Michieletti P, Wanless IR, Katz A (1994). Antimitochondrial antibody negative primary biliary cirrhosis: a distinct syndrome of autoimmune cholangitis.. Gut.

[19] Lacerda MA, Ludwig J, Dickson ER, Jorgensen RA, Lindor KD (1995). Antimitochondrial antibody-negative primary biliary cirrhosis.. Am J Gastroenterol.

[20] Kaplan MM, Cheng S, Price LL, Bonis PA (2004). A randomized controlled trial of colchicine plus ursodiol vs methotrexate plus ursodiol in primary biliary cirrhosis: ten-year results.. Hepatology.

[21] Lee J, Belanger A, Doucette JT (2007). Transplantation trends in primary biliary cirrhosis.. Clin Gastroenterol Hepatology.

[22] Akbar SM, Yamamoto K, Miyakawa H (2001). Peripheral blood T-cell responses to pyruvate dehydrogenase complex in primary biliary cirrhosis: role of antigenpresenting dendritic cells.. Eur J Clin Invest.

